# Supplementary material for: NIMA-related kinase 9 regulates the phosphorylation of the essential myosin light chain in the heart
Source: Nat Commun. 2022 Oct 20;13:6209. doi: 10.1038/s41467-022-33658-2 (PMC9585074; doi:10.1038/s41467-022-33658-2)
Supplement: Supplementary file 1 — Supplementary information file [file 41467_2022_33658_MOESM1_ESM.pdf]

Supplementary information

Supplementary Table 1: Patients characteristics. P-values refer to χ2, Wilcoxon and two-tailed unpaired Student's t-test for binary, skewed continuous and symmetrical continuous variables, respectively (HTX: n = 6, DCM: n = 11). *hs-TnT was measured for 6 DCM patients out of 11. All DCM patients included showed low TnT.

	**Ctrl HTX N=6**	**DCM N=11**	**p-value**
Age, mean (SD), years	62.3 (7.4)	62.3 (7.4)	0.640
Female sex, number (%)	1/6 (17%)	1/11 (9%)	0.643
LVEF, mean (SD), %	63.2 (3.2)	28.4 (11.7)	$3.844 \cdot 10^{-6}$
Dyspnoea, NYHA I, number (%)	6/6 (100%)	5/11 (45%)	0.025
Dyspnoea, NYHA II, number (%)	0/6 (0%)	4/11 (36%)	0.091
Dyspnoea, NYHA III, number (%)	0/6 (0%)	2/11 (18%)	0.266
Arterial hypertension, number (%)	4/6 (67%)	4/11 (36%)	0.232
Diabetes mellitus, number (%)	1/6 (17%)	0/11 (0%)	0.163
Mitral regurgitation, number (%)	1/6 (17%)	1/11 (9%)	0.643
Tricuspid regurgitation, number (%)	1/6 (17%)	1/11 (9%)	0.643
Mitral + Tricuspid regurgitation, number (%)	0/6 (0%)	2/11 (18%)	0.266
Mitral + Aorta regurgitation, number (%)	0/6 (0%)	2/11 (18%)	0.266
LVEDP, mean (SD), mmHg	19.3 (5.9)	25.1 (18.4)	0.474
RR systolic, mean (SD), mmHg	134.2 (12.8)	126.8 (15.0)	0.329
RR diastolic, mean (SD), mmHg	74.2 (11.1)	73.6 (10.3)	0.923
Heart rate, mean (SD), bpm	78.7 (9.1)	80.3 (11.8)	0.777
hs-TnT, median (1Q;3Q), pg/mL*	8.0 (6.0; 10.8)	9.8 (6.8; 9.8)	0.760
NTproBNP, median (1Q;3Q), ng/mL	87.0 (74.0; 136.0)	456.0 (117.0; 561.0)	0.099
Creatinine, mean (SD), mg/dL	1.2 (0.2)	1.1 (0.2)	0.391
GFP MDRD, mean (SD), mL/min/1.73qm	73.7 (14.3)	85.2 (14.8)	0.168
Urea, mean (SD), mg/dL	35.2 (7.9)	42.6 (21.9)	0.446
ACE inhibitor or ARB, number (%)	6/6 (100%)	11/11 (100%)	1.000
β-blocker, number (%)	2/6 (33%)	10/11 (91%)	0.013
Oral anticoagulants, number (%)	0/6 (0%)	4/11 (36%)	0.091
Loop diuretic, number (%)	1/6 (17%)	6/11 (55%)	0.129
Aspirin, number (%)	5/6 (83%)	2/11 (18%)	0.009
Statin, number (%)	6/6 (100%)	3/11 (27%)	0.004

Supplementary Figure 1

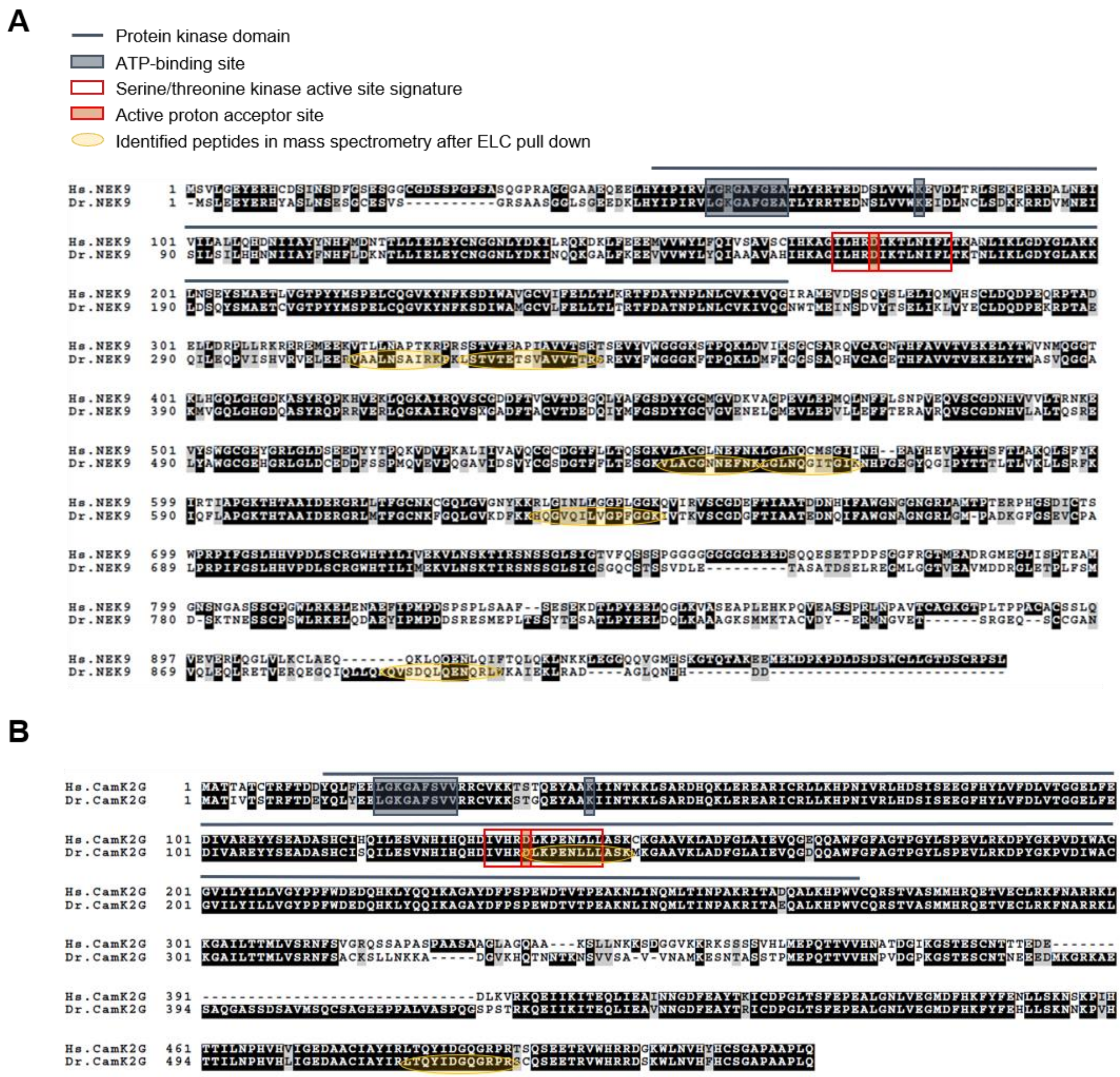

Supplementary Figure 1: Protein alignment of ELC interacting kinases. **A, B** Multiple protein sequence alignment of human (Hs.) versus piscine (Dr.) NEK9 (**A**) or CamK2G (**B**) performed by Clustal Omega Version 2.1. Protein domains are predicted by ExPASy-prosite (Release 2018_11). Peptides identified by mass spectrometry after ELC specific pull down are marked in yellow.

Supplementary Figure 2

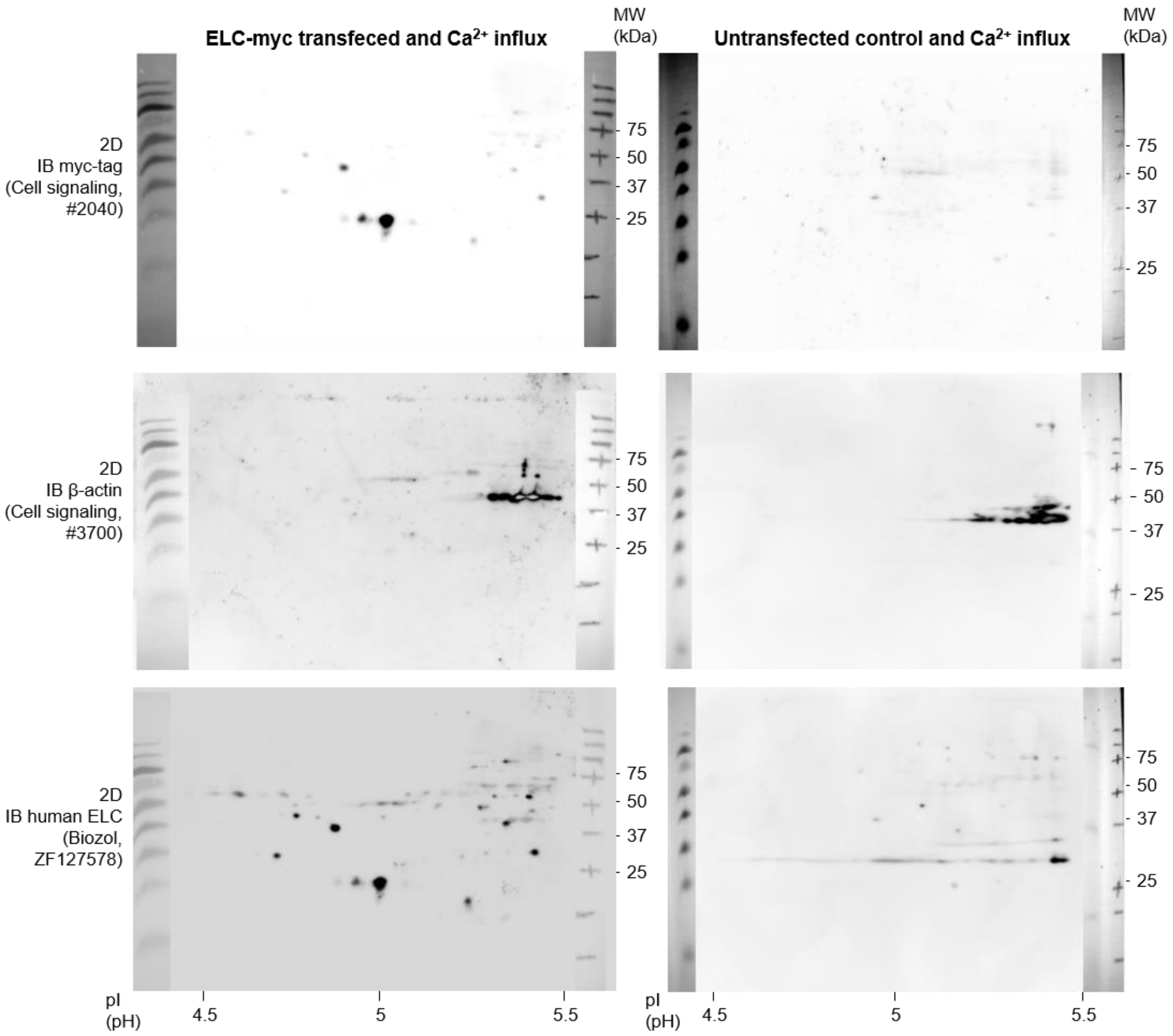

Supplementary Figure 2: Validation of ELC-phosphorylation pattern in-vitro. Myc-tagged human ELC was overexpressed in HEK cells and Ca^{2+} influx was obtained by ionophore stimulation. Untransfected, Ca^{2+}-treated cells served as control. 2D immunoblot was performed and blots of the same run were stained successively with myc-tag antibody (Cell signaling #2040), antibody against β-actin (Cell signaling #3700) and ELC specific antibody (Biozol ZF127578). One validation experiment to confirm results shown in Figure 3 C-E.

Supplementary Figure 3

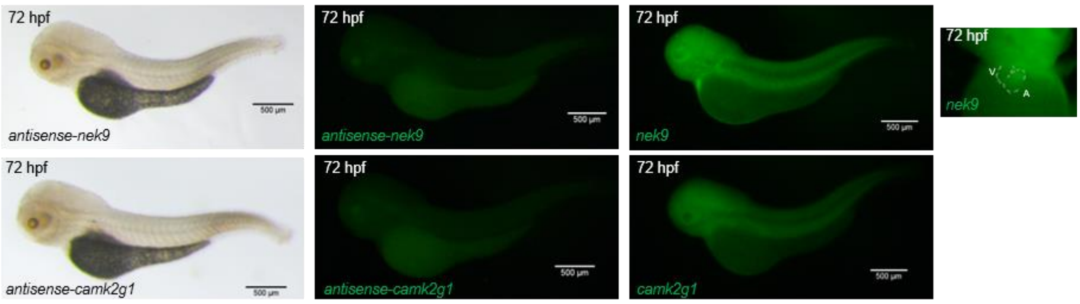

Supplementary Figure 3: Fluorescence in-situ hybridization of *nek9* or *camk2g1* in zebrafish embryos 72 hours post fertilization (hpf). Bright-field microscopy picture (**left**) and confocal microscopy picture (**right**) after hybridization with *nek9* or *camk2g1* specific RNA probes detected by fluorescein-labeled antibodies. Antisense RNA probes were used as negative control. One representative embryo was scanned out of 10 individuals.

Supplementary Figure 4

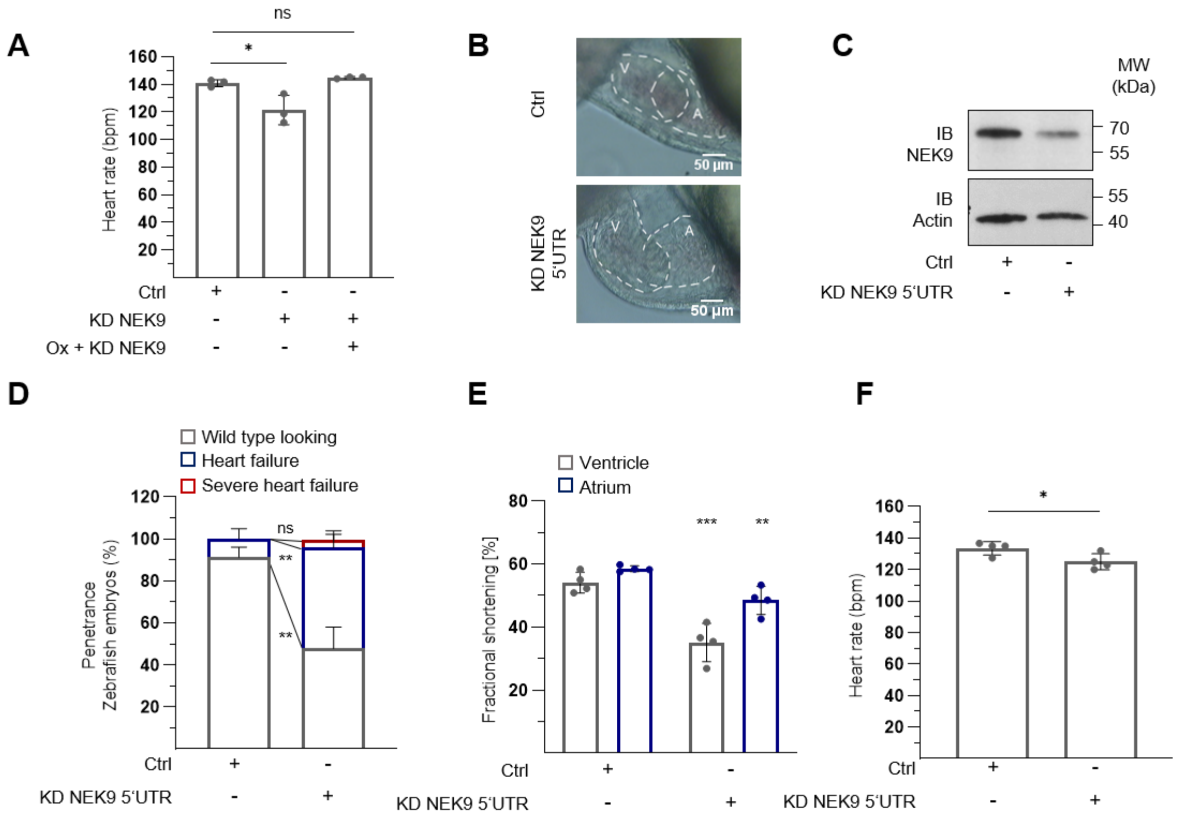

Supplementary Figure 4: Validation of NEK9 knockdown. **A** Heart rate in beats per min (bpm) at 72 hours post fertilization (hpf). Corresponding results are illustrated in Figure 4 A-C. Data are mean ± SD (Ctrl, n = 25, KD NEK9, n = 21, Ox + KD NEK9, n = 21 fish embryos out of 3 experiments). *P < 0.05 by the analysis of ordinary one-way ANOVA followed by Bonferroni's multiple comparisons test. **B** Lateral view of zebrafish embryos 72 hpf injected with nucleotides specifically binding the 5' UTR of zebrafish *nek9* (KD NEK9 5'UTR). **C** Validation of *nek9* knockdown by immunoblot (IB). **D** Penetrance of KD NEK9 5'UTR injection after 72 hpf. Data are mean ± SD (Ctrl, n = 360, KD NEK9 5'UTR, n = 298 fish embryos out of 4 experiments). **P < 0.01 by the analysis of two-tailed paired Student's t-test. **E** Fractional shortening (FS) at 72 hpf. **P < 0.01; ***P < 0.001 by the analysis of two-way ANOVA followed by Bonferroni's multiple comparisons test. **F** Heart rate in beats per min (bpm) at 72 hpf. *P < 0.05 by the analysis of two-tailed paired Student's t-test. For E and F data are mean ± SD (Ctrl, n = 38, KD NEK9 5'UTR, n = 42 fish embryos out of 4 experiments).

Supplementary Figure 5

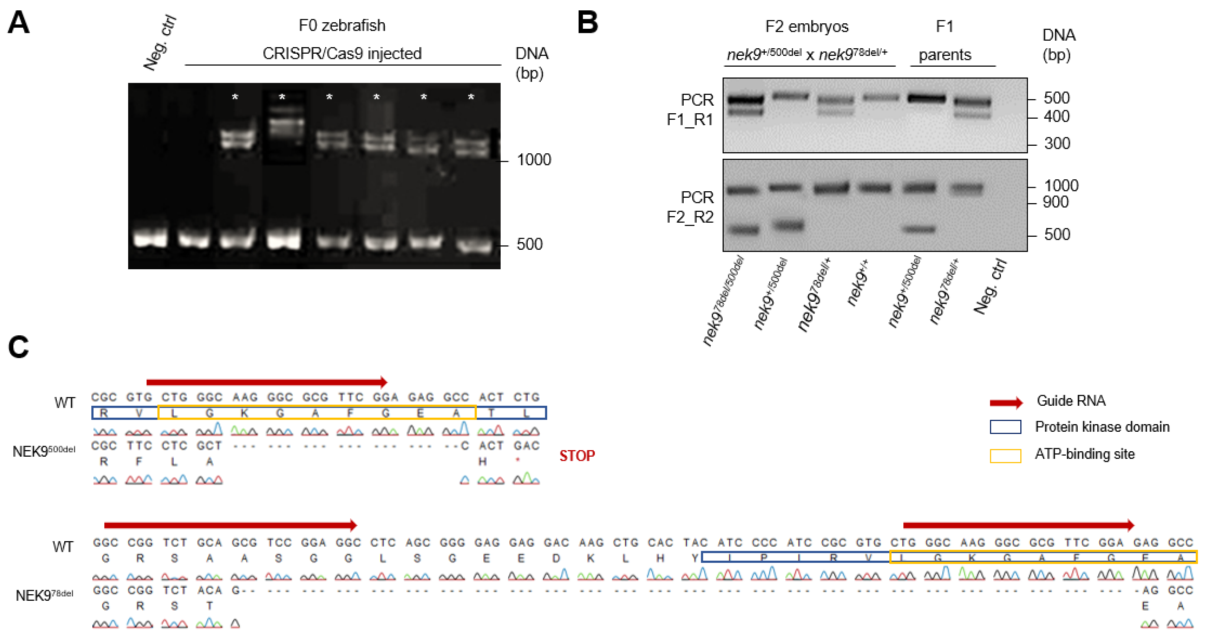

Supplementary Figure 5: Validation of CRISPR/Cas9 mediated knockout of *nek9* in zebrafish. Three guided RNAs (gRNA) targeting the genomic sequence of *nek9* in combination with CRISPR-associated (Cas9) protein were injected in zebrafish one-cell stage. Possible generated double-strand breaks leading to insertions or deletions were detected by a heteroduplex mobility assay.[1] In brief, the region of interest is amplified by PCR followed by denaturation of double-stranded molecules and annealing. Integrated mismatches result in incomplete annealing forming heteroduplex strands. The altered electrophoretic mobility of the heteroduplex strands compared to homoduplex bands are detected by polyacrylamide gel electrophoresis. The F0 generation was outcrossed with wild-type littermates and F1 offspring were genotyped. **A** Heteroduplex mortality assay used for the initial screen of *nek9* mutant alleles of F0 zebrafish 3-month post injection. DNA was isolated from adult zebrafish fin. Zebrafish with possible generated double-strand breaks are marked by stars (*). All adult zebrafish of the F0 generation were screened accordingly. **B** Heterozygous transgenic zebrafish $nek9^{78del/+}$ and $nek9^{+/500del}$ (F1 parents) were incrossed and offspring (F2 embryos) were analyzed by PCR amplification for specific genotyping. Primer pair F1_R1 is flanking the 78bp deletion and primer pair F2_R2 is flanking both deletions (78bp deletion and 500bp deletion). The two specific PCR markers were used for all genotype-phenotype-associations. **C** Alignment of sanger sequencing results of wild-type fish and heterozygous transgenic zebrafish $nek9^{78del/+}$ and $nek9^{+/500del}$ (F1 parents). *In silico* translation predicts consequences of the deletion on protein level. $NEK9^{500del}$ leads to a premature stop codon and $NEK9^{78del}$ results in loss of the ATP binding domain of NEK9.

Supplementary Figure 6

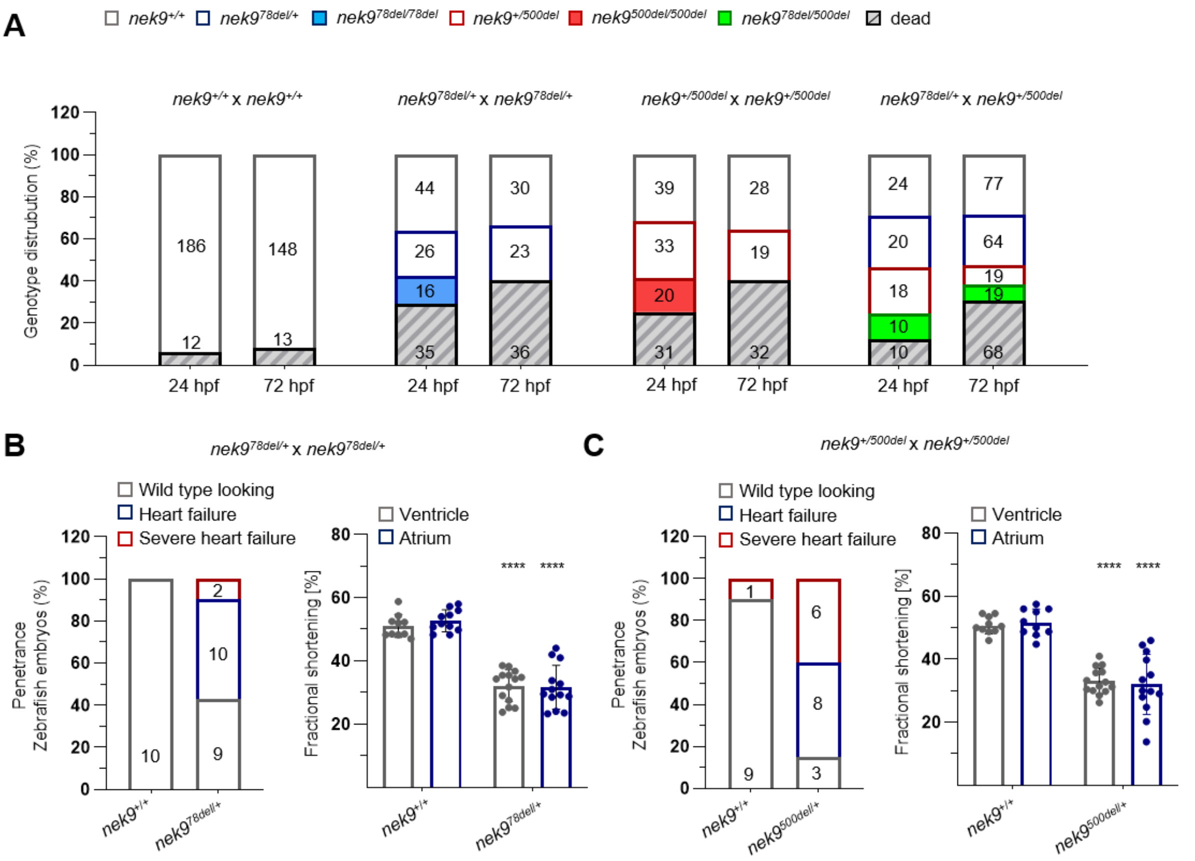

Supplementary Figure 6: Analysis of CRISPR/Cas9 generated transgenic zebrafish lines *nek9*^{78del} and *nek9*^{500del}. **A** Genotype distribution after incross (*nek9*$^{78del/+}$ x *nek9*$^{78del/+}$ or *nek9*$^{+/500del}$ x *nek9*$^{+/500del}$ or *nek9*$^{78del/+}$ x *nek9*$^{+/500del}$) of stable transgenic zebrafish lines at 24 or 72 hours post fertilization. Incross of wild type fish serves as control (*nek9*$^{+/+}$ x *nek9*$^{+/+}$). Mendelian ratios in % at 24 and 72 hpf (wild type /heterozygous /homozygous) are as follows: *nek9*$^{78del/+}$ x *nek9*$^{78del/+}$ (36/21/13), (34/26/0); *nek9*$^{+/500del}$ x *nek9*$^{+/500del}$ (32/27/16), (35/24/0); *nek9*$^{78del/+}$ x *nek9*$^{+/500del}$ (29/24/22/12) (23/19/13/8). **B, C** Phenotype analysis of transgenic zebrafish embryos at 72 hpf after incross of *nek9*$^{78del/+}$ x *nek9*$^{78del/+}$ **(B)** or incross of *nek9*$^{+/500del}$ *x nek9*$^{+/500del}$ **(C)**. Genotyping was performed after blinded phenotyping. Data are mean ± SD. The total number of analysed embryos is illustrated for each group. Statistical significance was calculated by a mixed effect model followed by Bonferroni's multiple comparisons test. ****$P < 0.0001$ are shown for the corresponding chamber of ctrl embryos.

Supplementary Figure 7

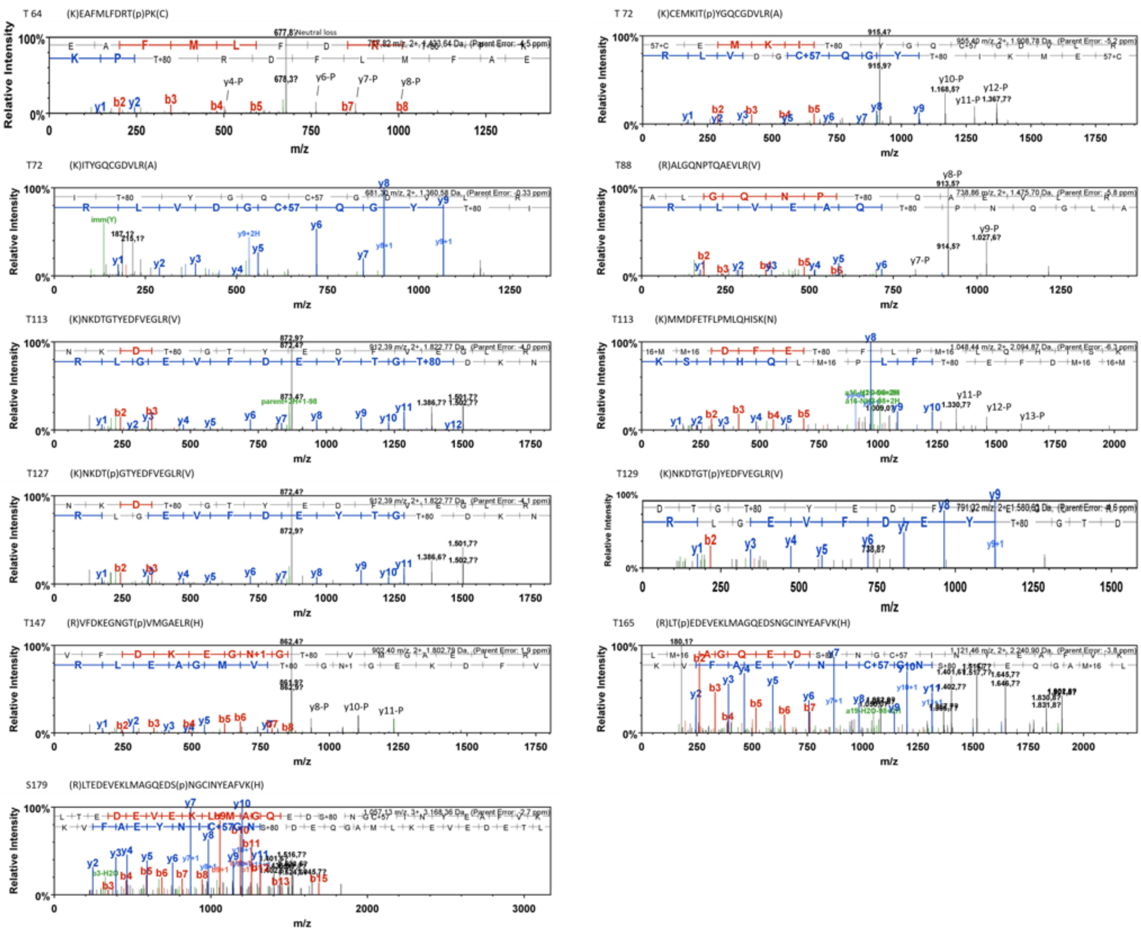

Supplementary Figure 7: Spectra for all nine ELC phospho peptides identified by mass spectrometry in spot arrays assigned to ventricular essential myosin light chain (vELC) (see Figure 7 A) in human left ventricular tissue (n = 6: DCM: n = 4; Ctrl: n = 2).

Supplementary Figure 8

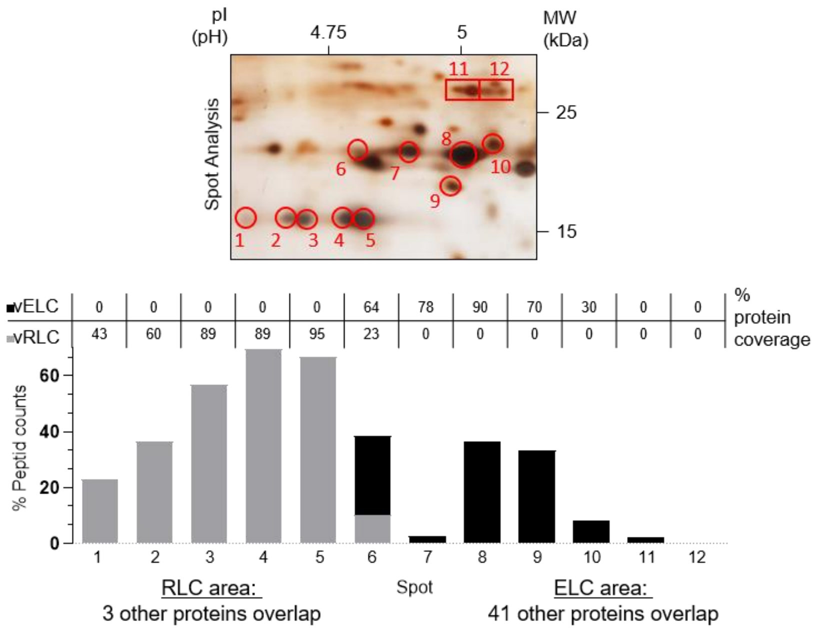

Supplementary Figure 8: Identification of protein spots in 2D page of human left ventricular tissue after Silver Stain (**upper panel**). Selected spot areas 1 to 12 were analyzed by liquid chromatography followed by mass spectrometry (LC-MS). Peptide counts uniquely assigned to ventricular essential myosin light chain (vELC) or regulatory myosin light chain (vRLC) are shown (**lower panel**). The mean percentage of the protein coverage of vELC and vRLC are given above (**table inset**).

Supplementary Figure 9

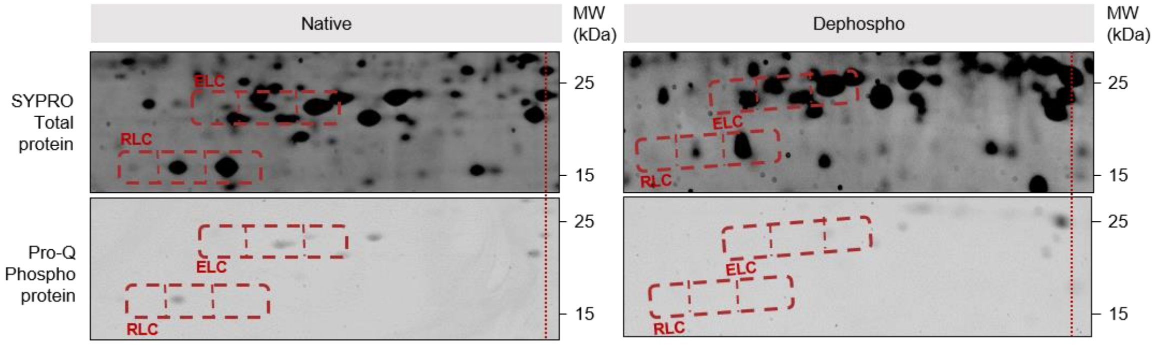

Supplementary Figure 9: Validation of dephosphorylation assay by using Pro-Q® Diamond Phosphoprotein Stain. Proteins of the same aliquots (see Figure 7 F) were separated by 2D electrophoresis and phosphorylated protein species were stained by Pro-Q Stain. SYPRO® Ruby Stain was used to visualize the total protein pattern. The experiment was done once to validate the results in Figure 3 C and Figure 8 C independently.

Supplementary Figure 10

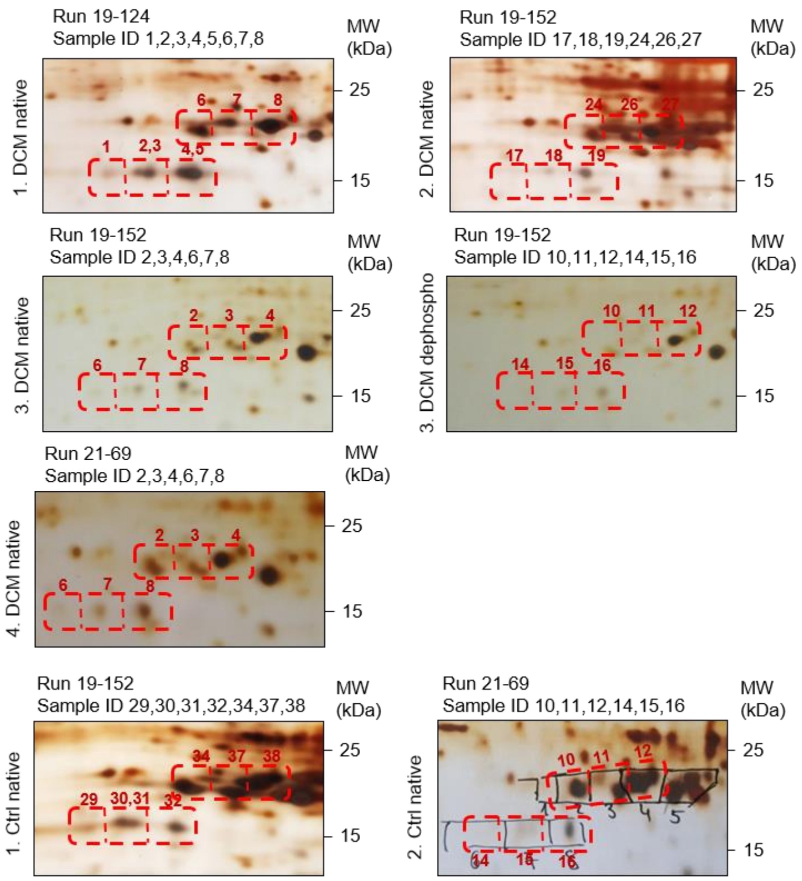

Supplementary Figure 10: 2D silver-stained gels of human LV tissue analyzed by mass spectrometry (MS). Spot areas corresponding to Figure 7 A-C and Figure 8 E, F are indicated in red boxes and MS sample IDs are added for each spot area. The mass spectrometry proteomics data have been deposited to the ProteomeXchange Consortium via the PRIDE[2] partner repository. Accession numbers are provided in the Data Availability Statement.

Supplementary references

1 Sorlien, E. L., Witucki, M. A. & Ogas, J. Efficient Production and Identification of CRISPR/Cas9-generated Gene Knockouts in the Model System Danio rerio. *J Vis Exp*, doi:10.3791/56969 (2018).

2 Perez-Riverol, Y. *et al.* The PRIDE database and related tools and resources in 2019: improving support for quantification data. *Nucleic Acids Res* **47**, D442-D450, doi:10.1093/nar/gky1106 (2019).
