# Supplementary material for: NIMA-related kinase 9 regulates the phosphorylation of the essential myosin light chain in the heart
Source: Nat Commun. 2022 Oct 20;13:6209. doi: 10.1038/s41467-022-33658-2 (PMC9585074; doi:10.1038/s41467-022-33658-2)
Supplement: Supplementary file 2 — Reporting Summary [file 41467_2022_33658_MOESM2_ESM.pdf]

nature research

Corresponding author(s): Benjamin Meder

Last updated by author(s): Jul 29, 2022

Reporting Summary

Nature Research wishes to improve the reproducibility of the work that we publish. This form provides structure for consistency and transparency in reporting. For further information on Nature Research policies, see Authors & Referees and the Editorial Policy Checklist .

Statistics

For all statistical analyses, confirm that the following items are present in the figure legend, table legend, main text, or Methods section.

n/a	Confirmed	
☐	☒	The exact sample size (*n*) for each experimental group/condition, given as a discrete number and unit of measurement
☐	☒	A statement on whether measurements were taken from distinct samples or whether the same sample was measured repeatedly
☐	☒	The statistical test(s) used AND whether they are one- or two-sided<br>*Only common tests should be described solely by name; describe more complex techniques in the Methods section.*
☒	☐	A description of all covariates tested
☐	☒	A description of any assumptions or corrections, such as tests of normality and adjustment for multiple comparisons
☐	☒	A full description of the statistical parameters including central tendency (e.g. means) or other basic estimates (e.g. regression coefficient) AND variation (e.g. standard deviation) or associated estimates of uncertainty (e.g. confidence intervals)
☐	☒	For null hypothesis testing, the test statistic (e.g. *F*, *t*, *r*) with confidence intervals, effect sizes, degrees of freedom and *P* value noted<br>*Give P values as exact values whenever suitable.*
☒	☐	For Bayesian analysis, information on the choice of priors and Markov chain Monte Carlo settings
☒	☐	For hierarchical and complex designs, identification of the appropriate level for tests and full reporting of outcomes
☒	☐	Estimates of effect sizes (e.g. Cohen's *d*, Pearson's *r*), indicating how they were calculated

Our web collection on statistics for biologists contains articles on many of the points above.

Software and code

Policy information about availability of computer code

Data collection	Proteome Discoverer 2.3 with SEQUEST HT: ISE (2.0.0.24, x64)
Data analysis	GraphPad Prism Version 5.0; ImageJ Version 1.51; Scaffold Version Scaffold_4.8.9; String interaction analysis, version 10.5; zebraFS software (www.benegfx.de); VirtualDub Version 1.7.

For manuscripts utilizing custom algorithms or software that are central to the research but not yet described in published literature, software must be made available to editors/reviewers. We strongly encourage code deposition in a community repository (e.g. GitHub). See the Nature Research guidelines for submitting code & software for further information.

Data

Policy information about availability of data

All manuscripts must include a data availability statement. This statement should provide the following information, where applicable:

- Accession codes, unique identifiers, or web links for publicly available datasets
- A list of figures that have associated raw data
- A description of any restrictions on data availability

The authors declare that the data supporting the findings of this study are available within the paper, its Supplementary Information files and Source Data file. The mass spectrometry proteomics data have been deposited to the ProteomeXchange Consortium via the PRIDE partner repository with the dataset identifier PXD016357, PXD034115 and PXD033908. The dataset with the project accession number PXD016357 contains the data presented in Figure 1 B-D and Supplemental Figure 1. The dataset with the accession number PXD034115 belongs to the APEX interaction screen presented in Figure 1 F. The dataset with the accession number PXD033908 contains the analysis of the myosin light chain migration pattern in 2D gels of human left ventricular proteins presented in Figure 7 A -C and Figure 8 E, F. Corresponding run and sample IDs are shown in Supplemental Figure 10. Phosphorylated peptides assigned to ELC are manually verified and representative spectra are presented in Supplemental Figure 7. The mRNA sequencing data shown in Figure 8 A are available at https://ccb-web.cs.uni-saarland.de/cms. Source Data are provided with this paper.

Field-specific reporting

Please select the one below that is the best fit for your research. If you are not sure, read the appropriate sections before making your selection.

- [x] Life sciences
- [ ] Behavioural & social sciences
- [ ] Ecological, evolutionary & environmental sciences

For a reference copy of the document with all sections, see nature.com/documents/nr-reporting-summary-flat.pdf

Life sciences study design

All studies must disclose on these points even when the disclosure is negative.

Sample size	Sample sizes for human material was limited by availablity of high quality tissue of well-phenotyped patients. For all zebrafish experiments (knockdown, knockout and sensitizing) the entire clutch of zebrafish embryos (offspring from one mating) was counted as one experiment.
Data exclusions	No data were excluded.
Replication	Experiments are replicated several times. The exact sample size of each experimental group is given for each figure.
Randomization	For human material: Inclusion criteria for DCM patients: LVEF <= 45%, availability of physical activity NYHA classification, NT-proBNP and hsTnT levels; inclusion crateria for HTX controls: LVEF >= 55%, NYHA I and hsTnT <= 51 ng/L.<br>For zebrafish experiments (kockout and sensitizing): A whole clutch of offspring was taken into account and the phenotype data were grouped by genotype. Genotyping was performed after blinded phenotyping. For knockdown experiments a clutch of zebrafish embryos was divided randomly into a control injected and anti-sense oligonucleotide injected group.
Blinding	The data were blinded through group allocation and data analysis. For zebrafish experiments (knockout and sensitizing): All embryos were phenotypically characterized at 24 or 72 hpf. Afterwards, the embryos were collected in a well plate with defined numbering/position for genotyping. The identified genotype was assigned to the previously documented phenotype for each fish and the penetrance was analyzed.

Reporting for specific materials, systems and methods

We require information from authors about some types of materials, experimental systems and methods used in many studies. Here, indicate whether each material, system or method listed is relevant to your study. If you are not sure if a list item applies to your research, read the appropriate section before selecting a response.

Materials & experimental systems

n/a	Involved in the study	
☐	☒	Antibodies
☐	☒	Eukaryotic cell lines
☒	☐	Palaeontology
☐	☒	Animals and other organisms
☐	☒	Human research participants
☒	☐	Clinical data

Methods

n/a	Involved in the study	
☒	☐	ChIP-seq
☒	☐	Flow cytometry
☒	☐	MRI-based neuroimaging

Antibodies

Antibodies used	rabbit polyclonal antibody against human ELC (Biozol, GeneTex, ZF127578), custom-made rabbit polyclonal antibody against piscine ELC (Eurogentec, immunization peptide: NH2-CAPVPETPKEPEVDLK-CONH2), mouse polyclonal antibody raised against the full-length protein of human CamK2G (MaxPab, Abnova, H00000818), mouse monoclonal antibody against human NEK9 (Abgent, AT3019a, clone name 1F6), mouse monoclonal antibody against actin (Sigma, #A2172, clone name 5C5), mouse monoclonal antibody against beta-actin (Sigma, #A5441, clone name AC-15), mouse monoclonal antibody against beta-actin (Cell signaling, #3700, clone name 8H10D10), mouse monoclonal antibody against flag-tag (Sigma, #F3165, clone name M2), rabbit polyclonal antibody against beta-tubulin (Abcam, #ab6046), rabbit polyclonal antibody against troponin I (Cell signaling #4002) and rabbit polyclonal antibody against phospho-troponin I (cardiac) (Ser23/24) (Cell signaling #4004), HRP-coupled secondary antibodies (Cell signaling, anti-mouse #7076, anti-rabbit #7074) and HRP-conjugated monoclonal antibody against myc-tag (Cell signaling, #2040, clone name 9B11). Antibody dilutions are provided within the Source Data file.
Validation	Quality control of the purified custom-made rabbit polyclonal antibody was performed by ELISA and SDS PAGE by Eurogentec. The Biozol rabbit polyclonal antibody against human ELC (GeneTex, ZF127578) was validated by titration of protein input amounts followed by IB and different exposure times (shown in the manuscript Figure 7). For all other primary antibodies recommanded concentrations in manufacturer's instructions were used.

Eukaryotic cell lines

Policy information about cell lines

Cell line source(s)	HEK293A cells (Life technologies, #R70507).
Authentication	Cell lines including safty documents were supplied by life technologies and no further technique for authentication was used.
Mycoplasma contamination	Cell lines were not further tested for mycoplasma contamination.
Commonly misidentified lines (See ICLAC register)	none.

Animals and other organisms

Policy information about studies involving animals; ARRIVE guidelines recommended for reporting animal research

Laboratory animals	Danio rerio; AB zebrafish line (European Zebrafish Research Center, ZDB-GENO-960809-7, https://zfin.org/ZDB-GENO-960809-7) and the transgenic zebrafish line lazy susan (The Zebrafish Information Network ID, ZDB-ALT-980203-503, https://zfin.org/ZDB-ALT-980203-503#summary). An entire clutch of zebrafish embryos (offspring from one mating) was used independently of sex. Analysis was performed at 24 or 72 hours post fertilization.
Wild animals	none.
Field-collected samples	none.
Ethics oversight	Care and breeding of Danio rerio was performed under institutional approval (Regierungspräsidium Karlsruhe, Baden-Württemberg, T52/17).

Note that full information on the approval of the study protocol must also be provided in the manuscript.

Human research participants

Policy information about studies involving human research participants

Population characteristics	All relevant patient characteristics are listed in Supplemental Table 1, including age, gender, diagnosis and medication.
Recruitment	The characterization of samples and patient data was performed within a central biobank approach (Department of Internal Medicine III, University Hospital Heidelberg). Participants have given written informed consent. A prerequisite for enrollment was leftover myocardial tissue from the routine diagnostic workflow. Human left ventricular tissue material of healthy controls have been used according to the protected health information (45 C.F.R. 164.514 e2) (bioserve) as well as the BCI informed consent F-641-5 (biochain).
Ethics oversight	Approved by Ethics Committee, Medical Faculty of Heidelberg (S390-2011).

Note that full information on the approval of the study protocol must also be provided in the manuscript.
