# Supplementary material for: NIMA-related kinase 9 regulates the phosphorylation of the essential myosin light chain in the heart
Source: Nat Commun. 2022 Oct 20;13:6209. doi: 10.1038/s41467-022-33658-2 (PMC9585074; doi:10.1038/s41467-022-33658-2)
Supplement: Supplementary file 6 — Description of Additional Supplementary Files [file 41467_2022_33658_MOESM6_ESM.pdf]

Description of Additional Supplementary Files

File name: Supplementary Movie 1

Description: Transient knockdown of nek9 in zebrafish embryos by injection with anti-sense oligonucleotides against zebrafish nek9 (MOnek9) compared to control injected embryos (MO-ctrl). Rescue experiments were performed by injection of nek9 mRNA before knockdown (mRNA nek9 +MO-nek9). Analysis at 72 hours post fertilization (hpf).

File name: Supplementary Movie 2

Description: CRISPR/Cas9 mediated transgenic mutant nek9 zebrafish embryos at 72 hours post fertilization (hpf). The nek9 78del allele leads to lack of the protein kinase domain of NEK9 and the nek9 500del allele results in loss of functional NEK9 protein by a premature stop codon. Heterozygous embryos (nek9(78del/+) and nek9(+/500del)) and a compound heterozygous embryo (nek9(78del/500del)) are compared to a wild type sibling (nek9(+/+)).

File name: Supplementary Movie 3

Description: Sensitizing experiment of nek9 in an ELC phospho-deficient genetic background lazy susan (laz(m647/m647). Low dose of anti-sense oligonucleotides against zebrafish nek9 (LD NEK9) or integrin-linked kinase (LD ILK) are injected in heterozygous lazy susan zebrafish embryos (laz(m647/+). Phenotype at 72 hours post fertilization (hpf).
